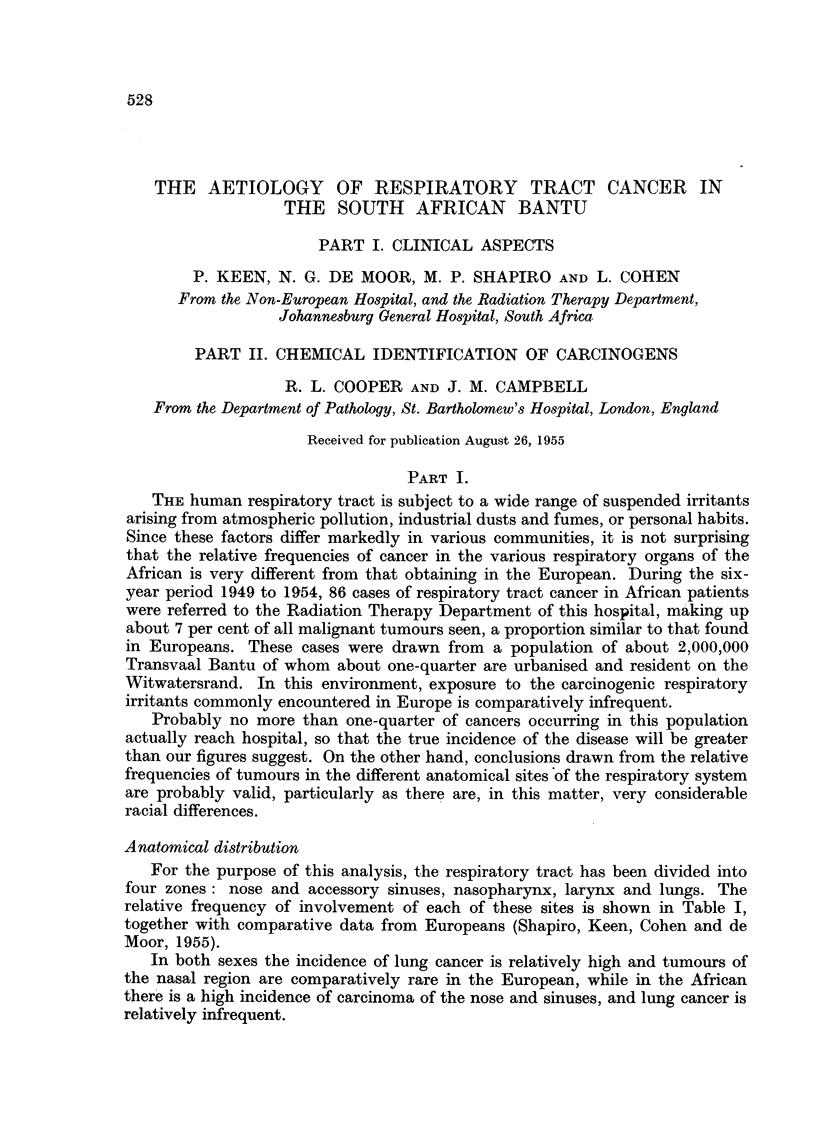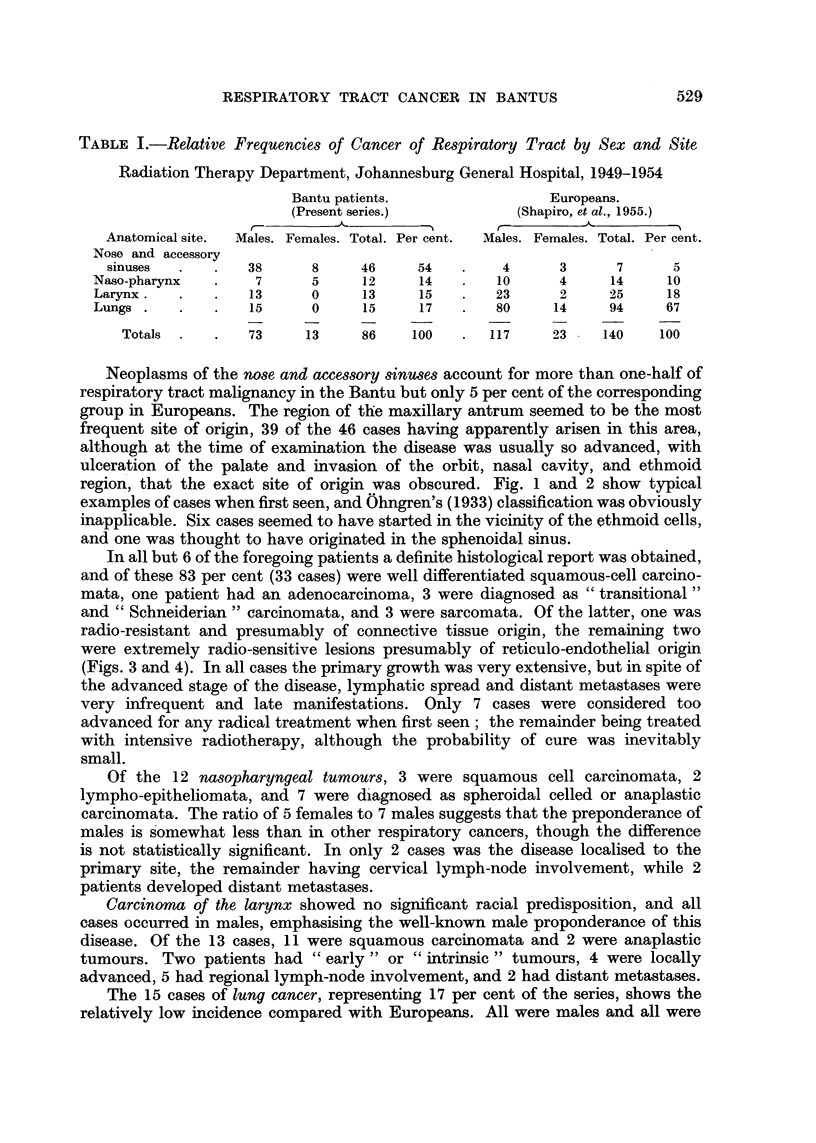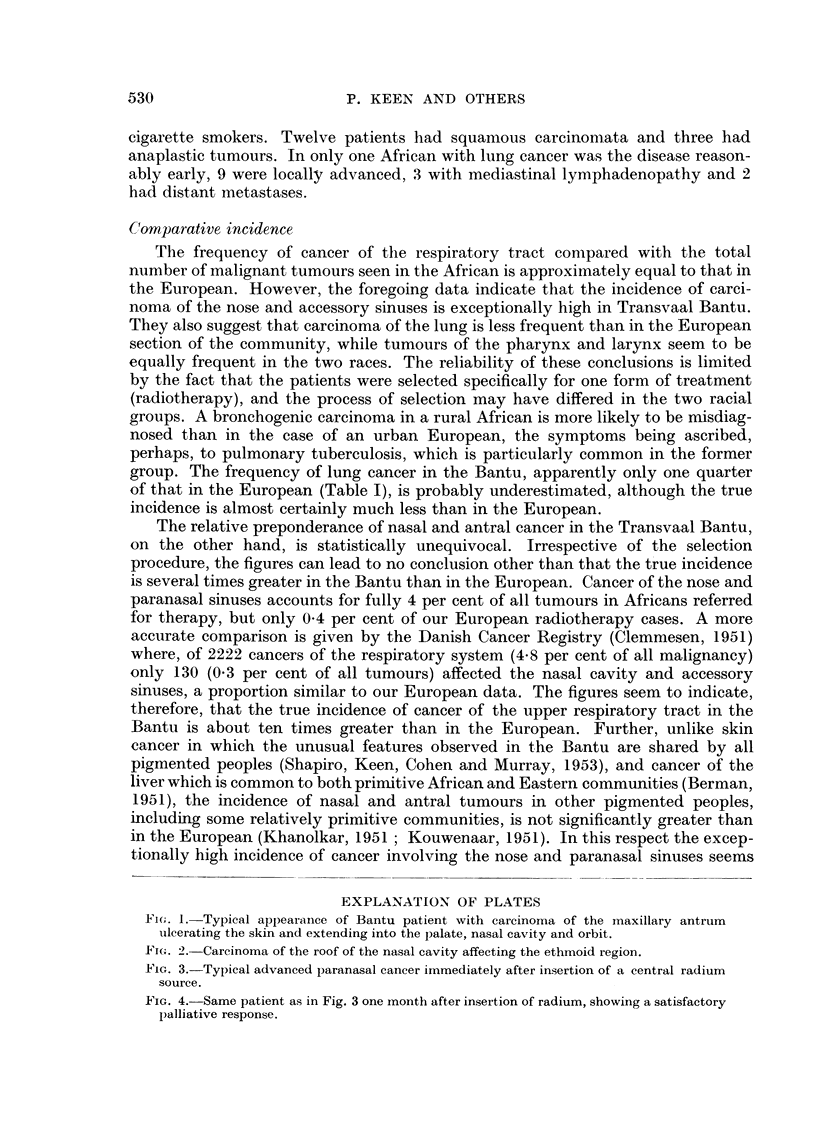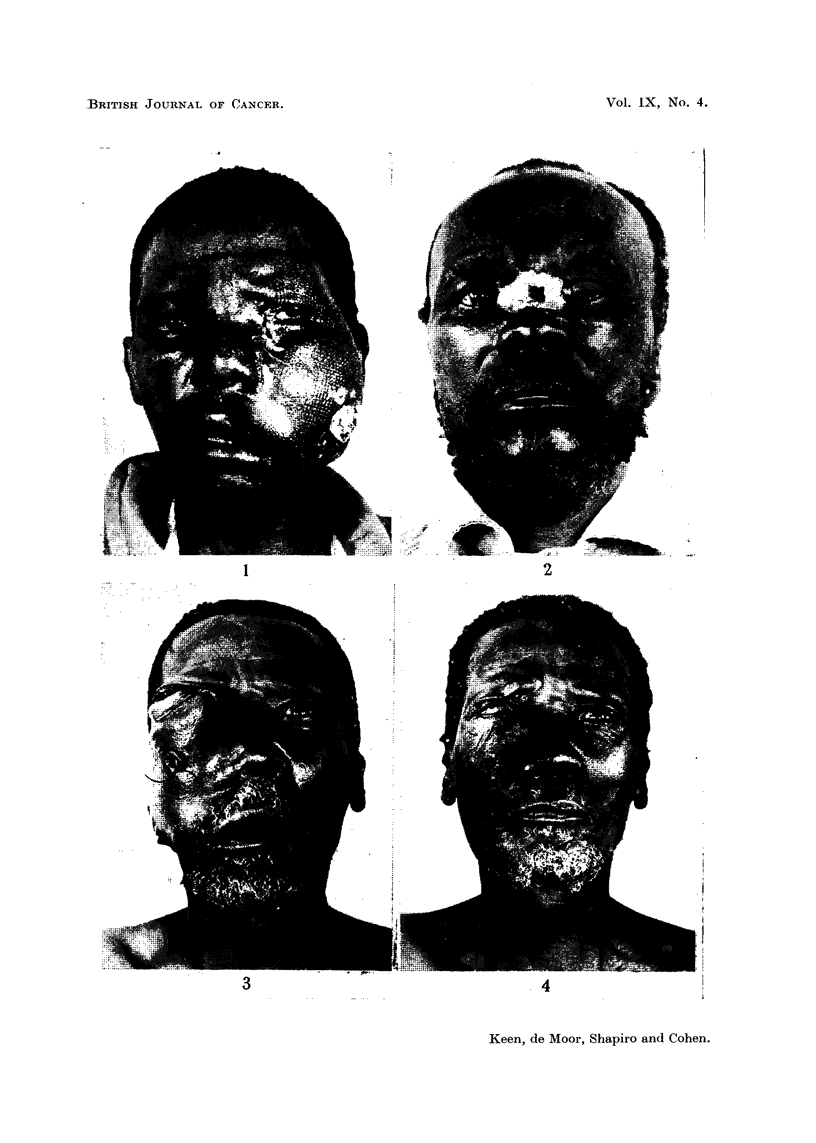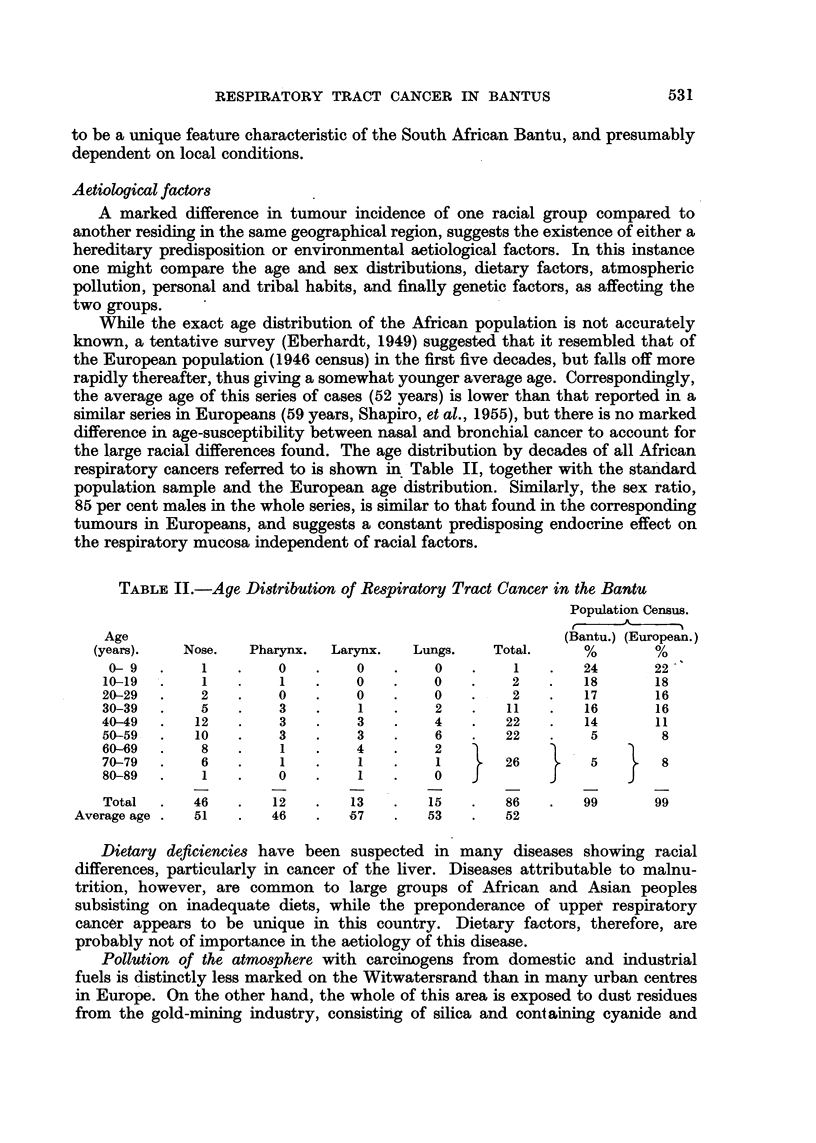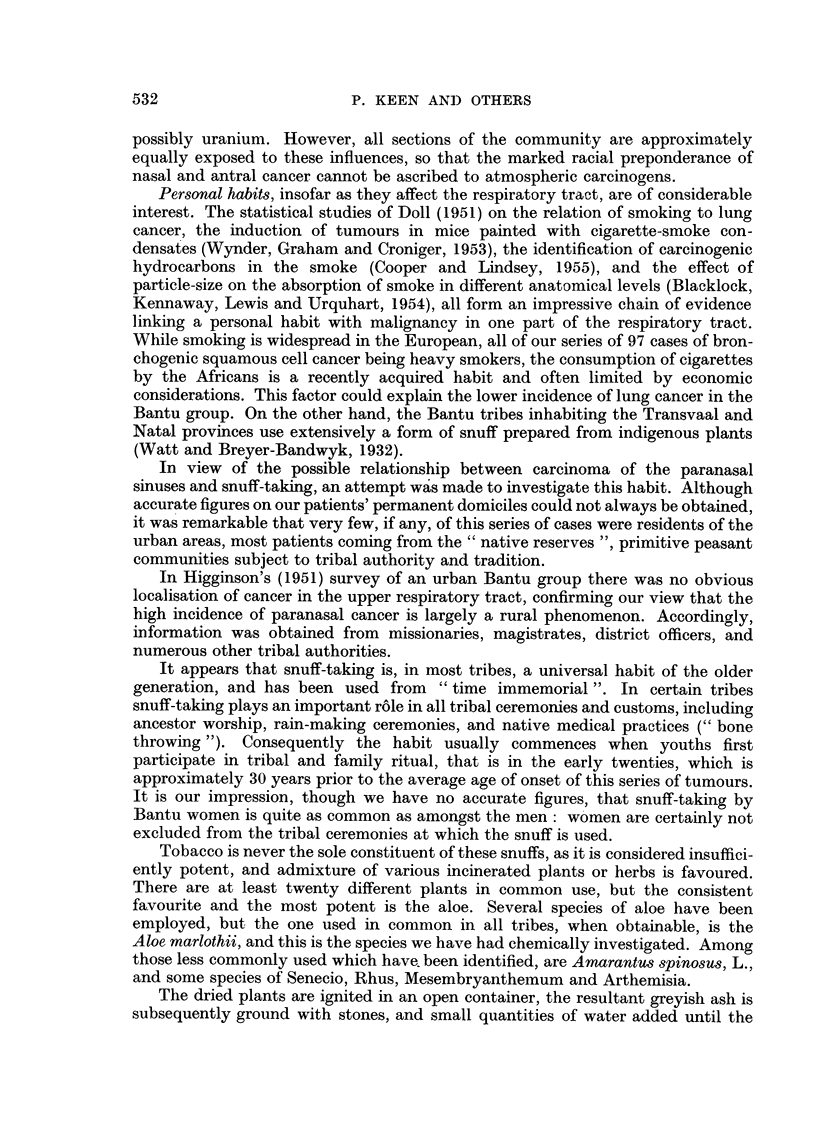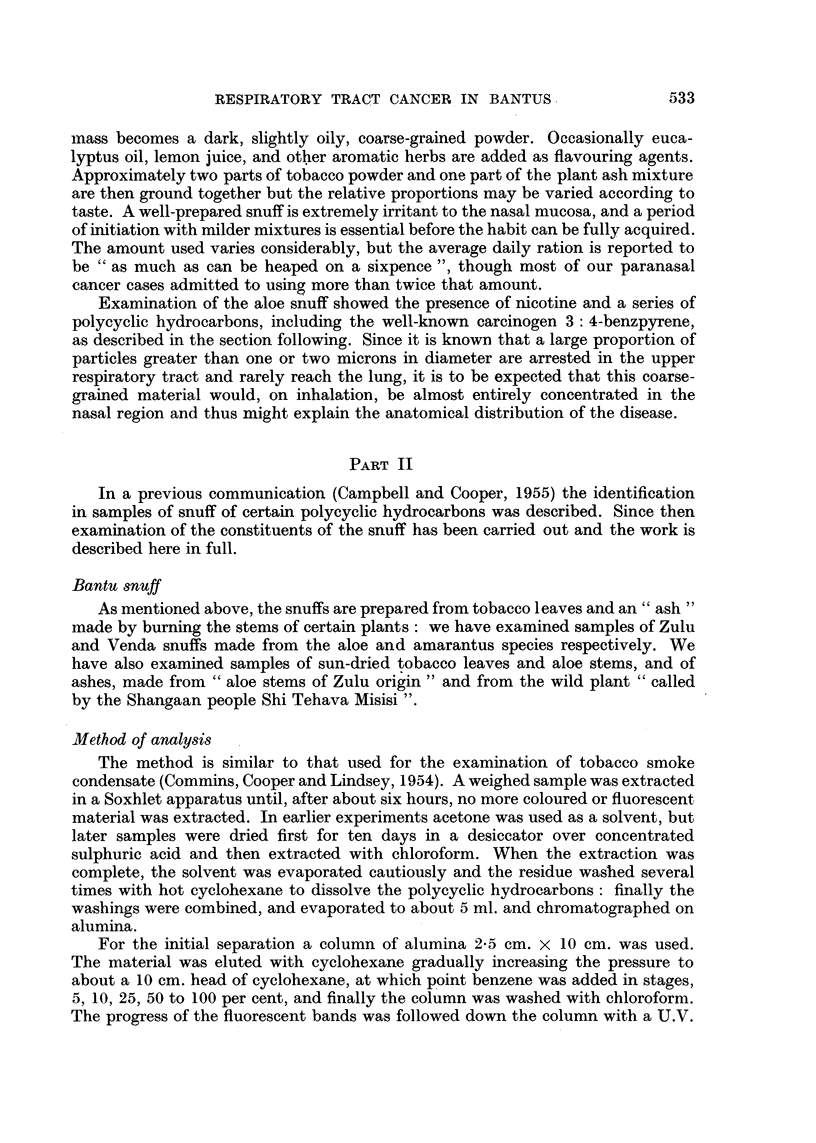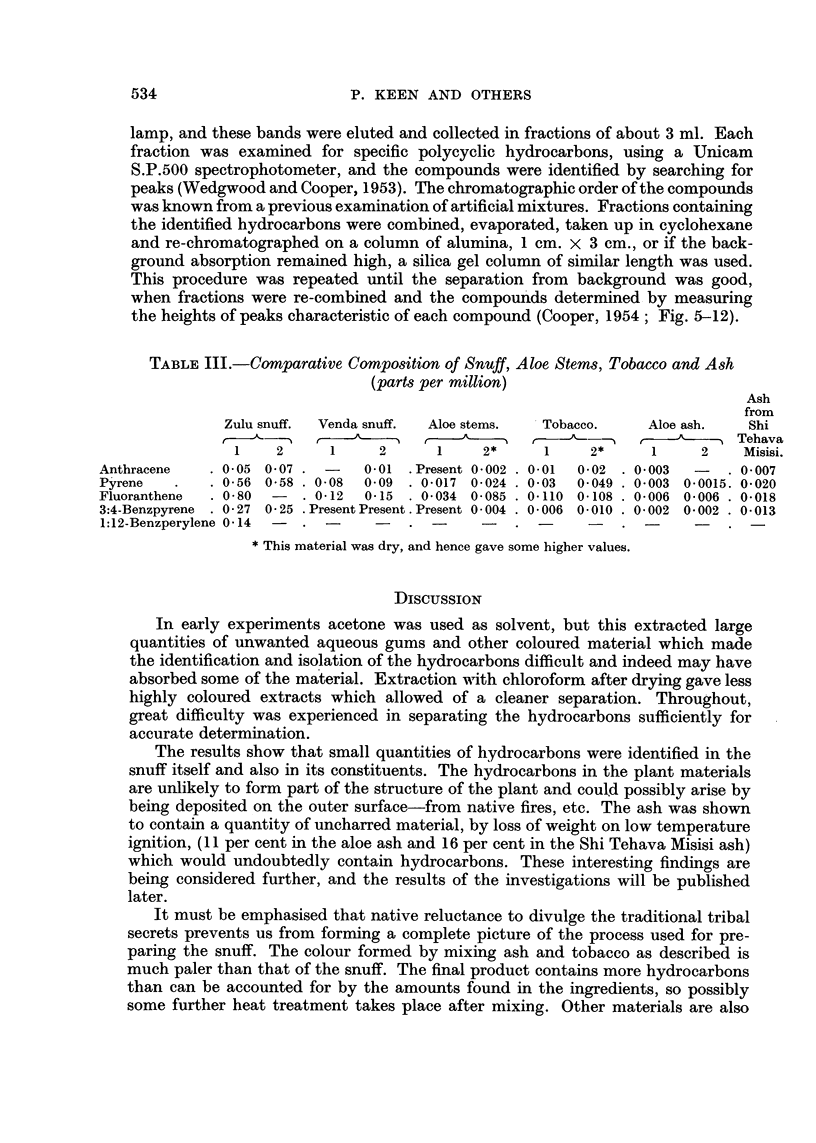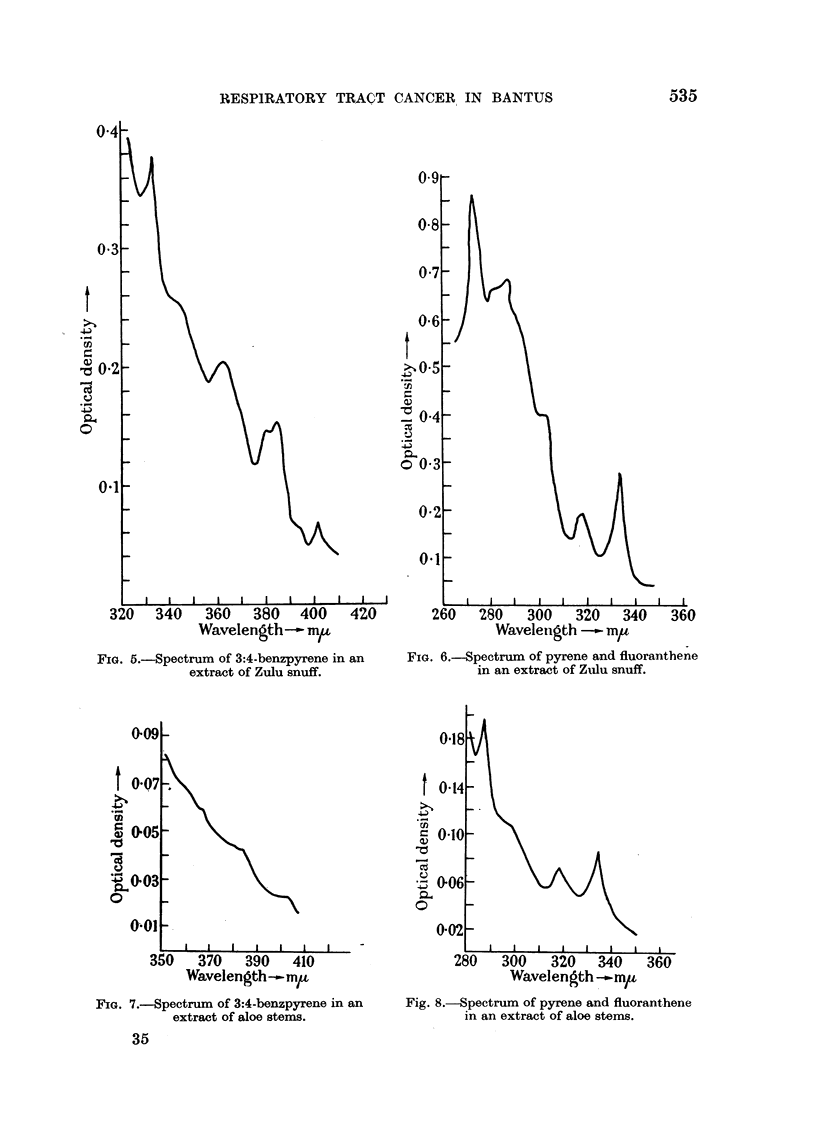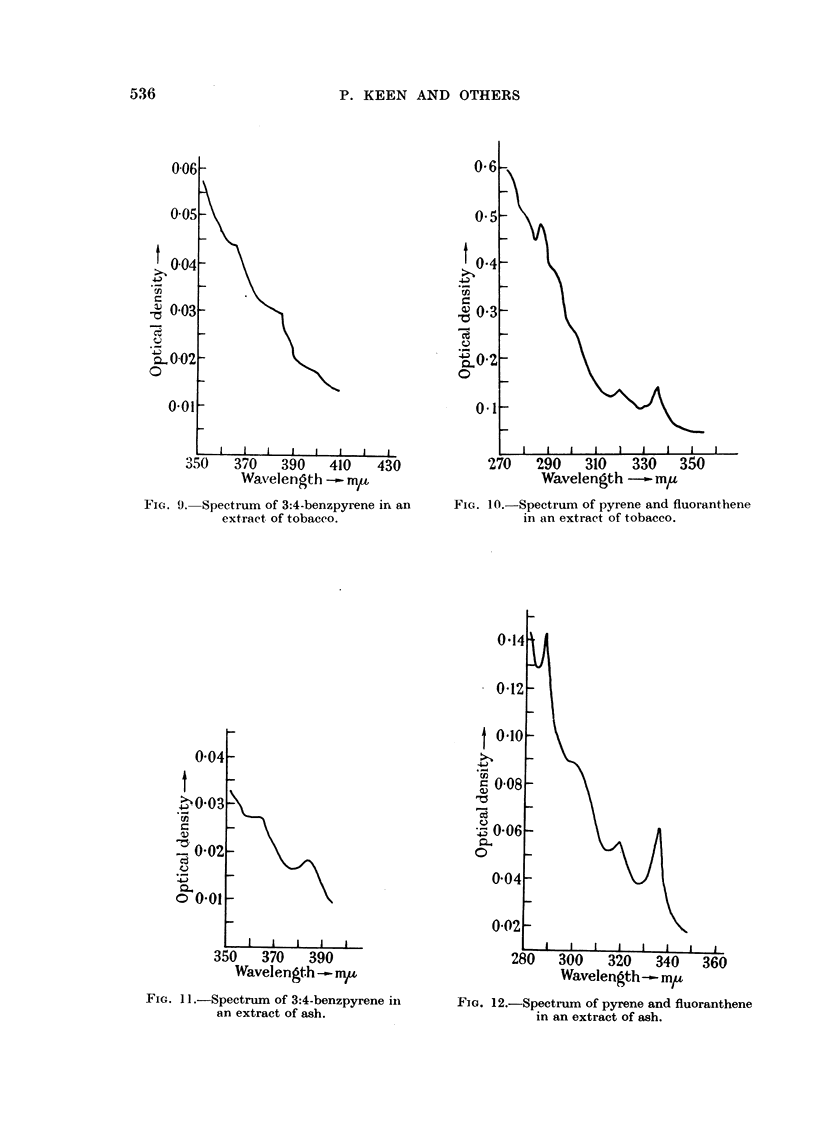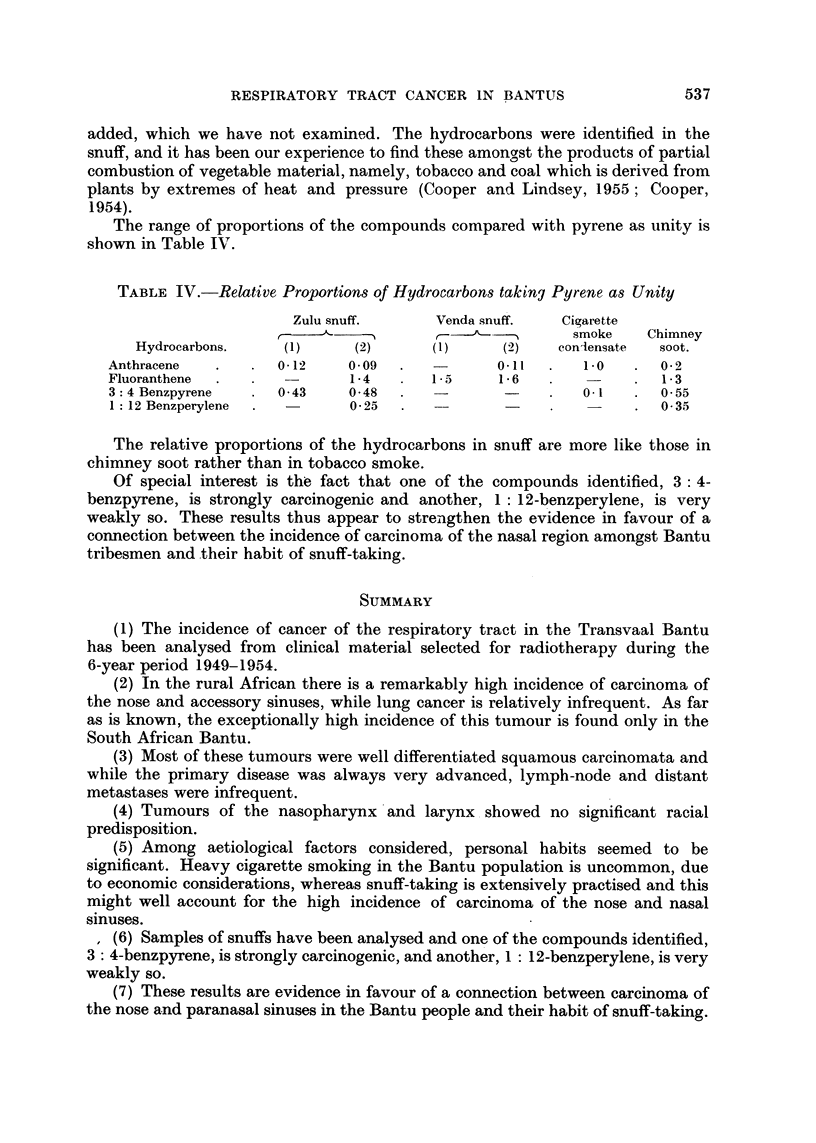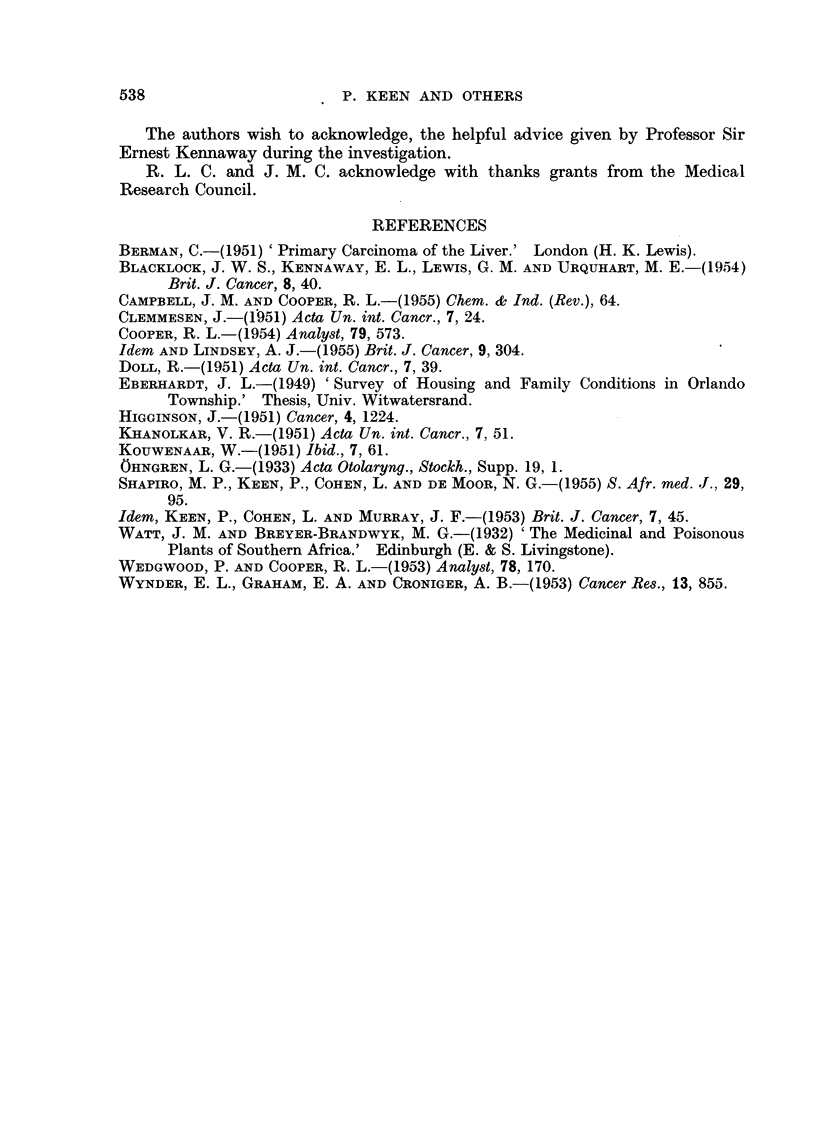# The Aetiology of Respiratory Tract Cancer in the South African Bantu

**DOI:** 10.1038/bjc.1955.54

**Published:** 1955-12

**Authors:** P. Keen, N. G. de Moor, M. P. Shapiro, L. Cohen, R. L. Cooper, J. M. Campbell

## Abstract

**Images:**


### THE AETIOLOGY OF RESPIRATORY TRACT CANCER IN THE SOUTH AFRICAN BANTU

### PART I. CLINICAL ASPECTS

P. KEEN, N. G. DE MOOR, M. P. SHAPIRO AND L. COHEN

*From the Non-European Hospital, and the Radiation Therapy Department, Johannesburg General Hospital, South Africa*

### PART II. CHEMICAL IDENTIFICATION OF CARCINOGENS

R. L. COOPER AND J. M. CAMPBELL

*From the Department of Pathology, St. Bartholomew's Hospital, London, England*

Received for publication August 26, 1955

### PART I.

THE human respiratory tract is subject to a wide range of suspended irritants arising from atmospheric pollution, industrial dusts and fumes, or personal habits. Since these factors differ markedly in various communities, it is not surprising that the relative frequencies of cancer in the various respiratory organs of the African is very different from that obtaining in the European. During the six-year period 1949 to 1954, 86 cases of respiratory tract cancer in African patients were referred to the Radiation Therapy Department of this hospital, making up about 7 per cent of all malignant tumours seen, a proportion similar to that found in Europeans. These cases were drawn from a population of about 2,000,000 Transvaal Bantu of whom about one-quarter are urbanised and resident on the Witwatersrand. In this environment, exposure to the carcinogenic respiratory irritants commonly encountered in Europe is comparatively infrequent.

Probably no more than one-quarter of cancers occurring in this population actually reach hospital, so that the true incidence of the disease will be greater than our figures suggest. On the other hand, conclusions drawn from the relative frequencies of tumours in the different anatomical sites of the respiratory system are probably valid, particularly as there are, in this matter, very considerable racial differences.

### *Anatomical distribution*

For the purpose of this analysis, the respiratory tract has been divided into four zones : nose and accessory sinuses, nasopharynx, larynx and lungs. The relative frequency of involvement of each of these sites is shown in Table I, together with comparative data from Europeans (Shapiro, Keen, Cohen and de Moor, 1955).

In both sexes the incidence of lung cancer is relatively high and tumours of the nasal region are comparatively rare in the European, while in the African there is a high incidence of carcinoma of the nose and sinuses, and lung cancer is relatively infrequent.

TABLE I.—*Relative Frequencies of Cancer of Respiratory Tract by Sex and Site*

Radiation Therapy Department, Johannesburg General Hospital, 1949–1954

| | Bantu patients. (Present series.) | | | | Europeans. (Shapiro, *et al.*, 1955.) | | | |
|---|---|---|---|---|---|---|---|---|
| Anatomical site. | Males. | Females. | Total. | Per cent. | Males. | Females. | Total. | Per cent. |
| Nose and accessory sinuses | 38 | 8 | 46 | 54 | 4 | 3 | 7 | 5 |
| Naso-pharynx | 7 | 5 | 12 | 14 | 10 | 4 | 14 | 10 |
| Larynx | 13 | 0 | 13 | 15 | 23 | 2 | 25 | 18 |
| Lungs | 15 | 0 | 15 | 17 | 80 | 14 | 94 | 67 |
| Totals | 73 | 13 | 86 | 100 | 117 | 23 | 140 | 100 |

Neoplasms of the *nose and accessory sinuses* account for more than one-half of respiratory tract malignancy in the Bantu but only 5 per cent of the corresponding group in Europeans. The region of the maxillary antrum seemed to be the most frequent site of origin, 39 of the 46 cases having apparently arisen in this area, although at the time of examination the disease was usually so advanced, with ulceration of the palate and invasion of the orbit, nasal cavity, and ethmoid region, that the exact site of origin was obscured. Fig. 1 and 2 show typical examples of cases when first seen, and Öhngren's (1933) classification was obviously inapplicable. Six cases seemed to have started in the vicinity of the ethmoid cells, and one was thought to have originated in the sphenoidal sinus.

In all but 6 of the foregoing patients a definite histological report was obtained, and of these 83 per cent (33 cases) were well differentiated squamous-cell carcinomata, one patient had an adenocarcinoma, 3 were diagnosed as " transitional " and " Schneiderian " carcinomata, and 3 were sarcomata. Of the latter, one was radio-resistant and presumably of connective tissue origin, the remaining two were extremely radio-sensitive lesions presumably of reticulo-endothelial origin (Figs. 3 and 4). In all cases the primary growth was very extensive, but in spite of the advanced stage of the disease, lymphatic spread and distant metastases were very infrequent and late manifestations. Only 7 cases were considered too advanced for any radical treatment when first seen ; the remainder being treated with intensive radiotherapy, although the probability of cure was inevitably small.

Of the 12 *nasopharyngeal tumours*, 3 were squamous cell carcinomata, 2 lympho-epitheliomata, and 7 were diagnosed as spheroidal celled or anaplastic carcinomata. The ratio of 5 females to 7 males suggests that the preponderance of males is somewhat less than in other respiratory cancers, though the difference is not statistically significant. In only 2 cases was the disease localised to the primary site, the remainder having cervical lymph-node involvement, while 2 patients developed distant metastases.

*Carcinoma of the larynx* showed no significant racial predisposition, and all cases occurred in males, emphasising the well-known male proponderance of this disease. Of the 13 cases, 11 were squamous carcinomata and 2 were anaplastic tumours. Two patients had " early " or " intrinsic " tumours, 4 were locally advanced, 5 had regional lymph-node involvement, and 2 had distant metastases.

The 15 cases of *lung cancer*, representing 17 per cent of the series, shows the relatively low incidence compared with Europeans. All were males and all were cigarette smokers. Twelve patients had squamous carcinomata and three had anaplastic tumours. In only one African with lung cancer was the disease reasonably early, 9 were locally advanced, 3 with mediastinal lymphadenopathy and 2 had distant metastases.

*Comparative incidence*

The frequency of cancer of the respiratory tract compared with the total number of malignant tumours seen in the African is approximately equal to that in the European. However, the foregoing data indicate that the incidence of carcinoma of the nose and accessory sinuses is exceptionally high in Transvaal Bantu. They also suggest that carcinoma of the lung is less frequent than in the European section of the community, while tumours of the pharynx and larynx seem to be equally frequent in the two races. The reliability of these conclusions is limited by the fact that the patients were selected specifically for one form of treatment (radiotherapy), and the process of selection may have differed in the two racial groups. A bronchogenic carcinoma in a rural African is more likely to be misdiagnosed than in the case of an urban European, the symptoms being ascribed, perhaps, to pulmonary tuberculosis, which is particularly common in the former group. The frequency of lung cancer in the Bantu, apparently only one quarter of that in the European (Table I), is probably underestimated, although the true incidence is almost certainly much less than in the European.

The relative preponderance of nasal and antral cancer in the Transvaal Bantu, on the other hand, is statistically unequivocal. Irrespective of the selection procedure, the figures can lead to no conclusion other than that the true incidence is several times greater in the Bantu than in the European. Cancer of the nose and paranasal sinuses accounts for fully 4 per cent of all tumours in Africans referred for therapy, but only 0·4 per cent of our European radiotherapy cases. A more accurate comparison is given by the Danish Cancer Registry (Clemmesen, 1951) where, of 2222 cancers of the respiratory system (4·8 per cent of all malignancy) only 130 (0·3 per cent of all tumours) affected the nasal cavity and accessory sinuses, a proportion similar to our European data. The figures seem to indicate, therefore, that the true incidence of cancer of the upper respiratory tract in the Bantu is about ten times greater than in the European. Further, unlike skin cancer in which the unusual features observed in the Bantu are shared by all pigmented peoples (Shapiro, Keen, Cohen and Murray, 1953), and cancer of the liver which is common to both primitive African and Eastern communities (Berman, 1951), the incidence of nasal and antral tumours in other pigmented peoples, including some relatively primitive communities, is not significantly greater than in the European (Khanolkar, 1951 ; Kouwenaar, 1951). In this respect the exceptionally high incidence of cancer involving the nose and paranasal sinuses seems

---

EXPLANATION OF PLATES

FIG. 1.—Typical appearance of Bantu patient with carcinoma of the maxillary antrum ulcerating the skin and extending into the palate, nasal cavity and orbit.

FIG. 2.—Carcinoma of the roof of the nasal cavity affecting the ethmoid region.

FIG. 3.—Typical advanced paranasal cancer immediately after insertion of a central radium source.

FIG. 4.—Same patient as in Fig. 3 one month after insertion of radium, showing a satisfactory palliative response.

1

2

3

4

Keen, de Moor, Shapiro and Cohen.

to be a unique feature characteristic of the South African Bantu, and presumably dependent on local conditions.

*Aetiological factors*

A marked difference in tumour incidence of one racial group compared to another residing in the same geographical region, suggests the existence of either a hereditary predisposition or environmental aetiological factors. In this instance one might compare the age and sex distributions, dietary factors, atmospheric pollution, personal and tribal habits, and finally genetic factors, as affecting the two groups.

While the exact age distribution of the African population is not accurately known, a tentative survey (Eberhardt, 1949) suggested that it resembled that of the European population (1946 census) in the first five decades, but falls off more rapidly thereafter, thus giving a somewhat younger average age. Correspondingly, the average age of this series of cases (52 years) is lower than that reported in a similar series in Europeans (59 years, Shapiro, *et al.*, 1955), but there is no marked difference in age-susceptibility between nasal and bronchial cancer to account for the large racial differences found. The age distribution by decades of all African respiratory cancers referred to is shown in Table II, together with the standard population sample and the European age distribution. Similarly, the sex ratio, 85 per cent males in the whole series, is similar to that found in the corresponding tumours in Europeans, and suggests a constant predisposing endocrine effect on the respiratory mucosa independent of racial factors.

TABLE II.—*Age Distribution of Respiratory Tract Cancer in the Bantu*

| Age (years). | Nose. | Pharynx. | Larynx. | Lungs. | Total. | Population Census. (Bantu.) % | Population Census. (European.) % |
|---|---|---|---|---|---|---|---|
| 0–9 | 1 | 0 | 0 | 0 | 1 | 24 | 22 |
| 10–19 | 1 | 1 | 0 | 0 | 2 | 18 | 18 |
| 20–29 | 2 | 0 | 0 | 0 | 2 | 17 | 16 |
| 30–39 | 5 | 3 | 1 | 2 | 11 | 16 | 16 |
| 40–49 | 12 | 3 | 3 | 4 | 22 | 14 | 11 |
| 50–59 | 10 | 3 | 3 | 6 | 22 | 5 | 8 |
| 60–69 | 8 | 1 | 4 | 2 | } 26 (60–89) | } 5 (60–89) | } 8 (60–89) |
| 70–79 | 6 | 1 | 1 | 1 | | | |
| 80–89 | 1 | 0 | 1 | 0 | | | |
| Total | 46 | 12 | 13 | 15 | 86 | 99 | 99 |
| Average age | 51 | 46 | 57 | 53 | 52 | | |

*Dietary deficiencies* have been suspected in many diseases showing racial differences, particularly in cancer of the liver. Diseases attributable to malnutrition, however, are common to large groups of African and Asian peoples subsisting on inadequate diets, while the preponderance of upper respiratory cancer appears to be unique in this country. Dietary factors, therefore, are probably not of importance in the aetiology of this disease.

*Pollution of the atmosphere* with carcinogens from domestic and industrial fuels is distinctly less marked on the Witwatersrand than in many urban centres in Europe. On the other hand, the whole of this area is exposed to dust residues from the gold-mining industry, consisting of silica and containing cyanide and possibly uranium. However, all sections of the community are approximately equally exposed to these influences, so that the marked racial preponderance of nasal and antral cancer cannot be ascribed to atmospheric carcinogens.

*Personal habits*, insofar as they affect the respiratory tract, are of considerable interest. The statistical studies of Doll (1951) on the relation of smoking to lung cancer, the induction of tumours in mice painted with cigarette-smoke condensates (Wynder, Graham and Croniger, 1953), the identification of carcinogenic hydrocarbons in the smoke (Cooper and Lindsey, 1955), and the effect of particle-size on the absorption of smoke in different anatomical levels (Blacklock, Kennaway, Lewis and Urquhart, 1954), all form an impressive chain of evidence linking a personal habit with malignancy in one part of the respiratory tract. While smoking is widespread in the European, all of our series of 97 cases of bronchogenic squamous cell cancer being heavy smokers, the consumption of cigarettes by the Africans is a recently acquired habit and often limited by economic considerations. This factor could explain the lower incidence of lung cancer in the Bantu group. On the other hand, the Bantu tribes inhabiting the Transvaal and Natal provinces use extensively a form of snuff prepared from indigenous plants (Watt and Breyer-Bandwyk, 1932).

In view of the possible relationship between carcinoma of the paranasal sinuses and snuff-taking, an attempt was made to investigate this habit. Although accurate figures on our patients' permanent domiciles could not always be obtained, it was remarkable that very few, if any, of this series of cases were residents of the urban areas, most patients coming from the " native reserves ", primitive peasant communities subject to tribal authority and tradition.

In Higginson's (1951) survey of an urban Bantu group there was no obvious localisation of cancer in the upper respiratory tract, confirming our view that the high incidence of paranasal cancer is largely a rural phenomenon. Accordingly, information was obtained from missionaries, magistrates, district officers, and numerous other tribal authorities.

It appears that snuff-taking is, in most tribes, a universal habit of the older generation, and has been used from " time immemorial ". In certain tribes snuff-taking plays an important rôle in all tribal ceremonies and customs, including ancestor worship, rain-making ceremonies, and native medical practices (" bone throwing "). Consequently the habit usually commences when youths first participate in tribal and family ritual, that is in the early twenties, which is approximately 30 years prior to the average age of onset of this series of tumours. It is our impression, though we have no accurate figures, that snuff-taking by Bantu women is quite as common as amongst the men : women are certainly not excluded from the tribal ceremonies at which the snuff is used.

Tobacco is never the sole constituent of these snuffs, as it is considered insufficiently potent, and admixture of various incinerated plants or herbs is favoured. There are at least twenty different plants in common use, but the consistent favourite and the most potent is the aloe. Several species of aloe have been employed, but the one used in common in all tribes, when obtainable, is the *Aloe marlothii*, and this is the species we have had chemically investigated. Among those less commonly used which have been identified, are *Amarantus spinosus*, L., and some species of Senecio, Rhus, Mesembryanthemum and Arthemisia.

The dried plants are ignited in an open container, the resultant greyish ash is subsequently ground with stones, and small quantities of water added until the mass becomes a dark, slightly oily, coarse-grained powder. Occasionally eucalyptus oil, lemon juice, and other aromatic herbs are added as flavouring agents. Approximately two parts of tobacco powder and one part of the plant ash mixture are then ground together but the relative proportions may be varied according to taste. A well-prepared snuff is extremely irritant to the nasal mucosa, and a period of initiation with milder mixtures is essential before the habit can be fully acquired. The amount used varies considerably, but the average daily ration is reported to be " as much as can be heaped on a sixpence ", though most of our paranasal cancer cases admitted to using more than twice that amount.

Examination of the aloe snuff showed the presence of nicotine and a series of polycyclic hydrocarbons, including the well-known carcinogen 3 : 4-benzpyrene, as described in the section following. Since it is known that a large proportion of particles greater than one or two microns in diameter are arrested in the upper respiratory tract and rarely reach the lung, it is to be expected that this coarse-grained material would, on inhalation, be almost entirely concentrated in the nasal region and thus might explain the anatomical distribution of the disease.

### Part II

In a previous communication (Campbell and Cooper, 1955) the identification in samples of snuff of certain polycyclic hydrocarbons was described. Since then examination of the constituents of the snuff has been carried out and the work is described here in full.

### *Bantu snuff*

As mentioned above, the snuffs are prepared from tobacco leaves and an " ash " made by burning the stems of certain plants : we have examined samples of Zulu and Venda snuffs made from the aloe and amarantus species respectively. We have also examined samples of sun-dried tobacco leaves and aloe stems, and of ashes, made from " aloe stems of Zulu origin " and from the wild plant " called by the Shangaan people Shi Tehava Misisi ".

### *Method of analysis*

The method is similar to that used for the examination of tobacco smoke condensate (Commins, Cooper and Lindsey, 1954). A weighed sample was extracted in a Soxhlet apparatus until, after about six hours, no more coloured or fluorescent material was extracted. In earlier experiments acetone was used as a solvent, but later samples were dried first for ten days in a desiccator over concentrated sulphuric acid and then extracted with chloroform. When the extraction was complete, the solvent was evaporated cautiously and the residue washed several times with hot cyclohexane to dissolve the polycyclic hydrocarbons : finally the washings were combined, and evaporated to about 5 ml. and chromatographed on alumina.

For the initial separation a column of alumina 2·5 cm. × 10 cm. was used. The material was eluted with cyclohexane gradually increasing the pressure to about a 10 cm. head of cyclohexane, at which point benzene was added in stages, 5, 10, 25, 50 to 100 per cent, and finally the column was washed with chloroform. The progress of the fluorescent bands was followed down the column with a U.V.

lamp, and these bands were eluted and collected in fractions of about 3 ml. Each fraction was examined for specific polycyclic hydrocarbons, using a Unicam S.P.500 spectrophotometer, and the compounds were identified by searching for peaks (Wedgwood and Cooper, 1953). The chromatographic order of the compounds was known from a previous examination of artificial mixtures. Fractions containing the identified hydrocarbons were combined, evaporated, taken up in cyclohexane and re-chromatographed on a column of alumina, 1 cm. × 3 cm., or if the background absorption remained high, a silica gel column of similar length was used. This procedure was repeated until the separation from background was good, when fractions were re-combined and the compounds determined by measuring the heights of peaks characteristic of each compound (Cooper, 1954 ; Fig. 5–12).

TABLE III.—*Comparative Composition of Snuff, Aloe Stems, Tobacco and Ash (parts per million)*

| | Zulu snuff. | | Venda snuff. | | Aloe stems. | | Tobacco. | | Aloe ash. | | Ash from Shi Tehava Misisi. |
|---|---|---|---|---|---|---|---|---|---|---|---|
| | 1 | 2 | 1 | 2 | 1 | 2* | 1 | 2* | 1 | 2 | |
| Anthracene | 0·05 | 0·07 | — | 0·01 | Present | 0·002 | 0·01 | 0·02 | 0·003 | — | 0·007 |
| Pyrene | 0·56 | 0·58 | 0·08 | 0·09 | 0·017 | 0·024 | 0·03 | 0·049 | 0·003 | 0·0015 | 0·020 |
| Fluoranthene | 0·80 | — | 0·12 | 0·15 | 0·034 | 0·085 | 0·110 | 0·108 | 0·006 | 0·006 | 0·018 |
| 3:4-Benzpyrene | 0·27 | 0·25 | Present | Present | Present | 0·004 | 0·006 | 0·010 | 0·002 | 0·002 | 0·013 |
| 1:12-Benzperylene | 0·14 | — | — | — | — | — | — | — | — | — | — |

* This material was dry, and hence gave some higher values.

### DISCUSSION

In early experiments acetone was used as solvent, but this extracted large quantities of unwanted aqueous gums and other coloured material which made the identification and isolation of the hydrocarbons difficult and indeed may have absorbed some of the material. Extraction with chloroform after drying gave less highly coloured extracts which allowed of a cleaner separation. Throughout, great difficulty was experienced in separating the hydrocarbons sufficiently for accurate determination.

The results show that small quantities of hydrocarbons were identified in the snuff itself and also in its constituents. The hydrocarbons in the plant materials are unlikely to form part of the structure of the plant and could possibly arise by being deposited on the outer surface—from native fires, etc. The ash was shown to contain a quantity of uncharred material, by loss of weight on low temperature ignition, (11 per cent in the aloe ash and 16 per cent in the Shi Tehava Misisi ash) which would undoubtedly contain hydrocarbons. These interesting findings are being considered further, and the results of the investigations will be published later.

It must be emphasised that native reluctance to divulge the traditional tribal secrets prevents us from forming a complete picture of the process used for preparing the snuff. The colour formed by mixing ash and tobacco as described is much paler than that of the snuff. The final product contains more hydrocarbons than can be accounted for by the amounts found in the ingredients, so possibly some further heat treatment takes place after mixing. Other materials are also

FIG. 5.—Spectrum of 3:4-benzpyrene in an extract of Zulu snuff.

FIG. 6.—Spectrum of pyrene and fluoranthene in an extract of Zulu snuff.

FIG. 7.—Spectrum of 3:4-benzpyrene in an extract of aloe stems.

Fig. 8.—Spectrum of pyrene and fluoranthene in an extract of aloe stems.

FIG. 9.—Spectrum of 3:4-benzpyrene in an extract of tobacco.

FIG. 10.—Spectrum of pyrene and fluoranthene in an extract of tobacco.

FIG. 11.—Spectrum of 3:4-benzpyrene in an extract of ash.

FIG. 12.—Spectrum of pyrene and fluoranthene in an extract of ash.

added, which we have not examined. The hydrocarbons were identified in the snuff, and it has been our experience to find these amongst the products of partial combustion of vegetable material, namely, tobacco and coal which is derived from plants by extremes of heat and pressure (Cooper and Lindsey, 1955; Cooper, 1954).

The range of proportions of the compounds compared with pyrene as unity is shown in Table IV.

TABLE IV.—*Relative Proportions of Hydrocarbons taking Pyrene as Unity*

| Hydrocarbons. | Zulu snuff. (1) | Zulu snuff. (2) | Venda snuff. (1) | Venda snuff. (2) | Cigarette smoke condensate | Chimney soot. |
|---|---|---|---|---|---|---|
| Anthracene | 0·12 | 0·09 | — | 0·11 | 1·0 | 0·2 |
| Fluoranthene | — | 1·4 | 1·5 | 1·6 | — | 1·3 |
| 3 : 4 Benzpyrene | 0·43 | 0·48 | — | — | 0·1 | 0·55 |
| 1 : 12 Benzperylene | — | 0·25 | — | — | — | 0·35 |

The relative proportions of the hydrocarbons in snuff are more like those in chimney soot rather than in tobacco smoke.

Of special interest is the fact that one of the compounds identified, 3 : 4-benzpyrene, is strongly carcinogenic and another, 1 : 12-benzperylene, is very weakly so. These results thus appear to strengthen the evidence in favour of a connection between the incidence of carcinoma of the nasal region amongst Bantu tribesmen and their habit of snuff-taking.

### SUMMARY

(1) The incidence of cancer of the respiratory tract in the Transvaal Bantu has been analysed from clinical material selected for radiotherapy during the 6-year period 1949–1954.

(2) In the rural African there is a remarkably high incidence of carcinoma of the nose and accessory sinuses, while lung cancer is relatively infrequent. As far as is known, the exceptionally high incidence of this tumour is found only in the South African Bantu.

(3) Most of these tumours were well differentiated squamous carcinomata and while the primary disease was always very advanced, lymph-node and distant metastases were infrequent.

(4) Tumours of the nasopharynx and larynx showed no significant racial predisposition.

(5) Among aetiological factors considered, personal habits seemed to be significant. Heavy cigarette smoking in the Bantu population is uncommon, due to economic considerations, whereas snuff-taking is extensively practised and this might well account for the high incidence of carcinoma of the nose and nasal sinuses.

(6) Samples of snuffs have been analysed and one of the compounds identified, 3 : 4-benzpyrene, is strongly carcinogenic, and another, 1 : 12-benzperylene, is very weakly so.

(7) These results are evidence in favour of a connection between carcinoma of the nose and paranasal sinuses in the Bantu people and their habit of snuff-taking.

The authors wish to acknowledge, the helpful advice given by Professor Sir Ernest Kennaway during the investigation.

R. L. C. and J. M. C. acknowledge with thanks grants from the Medical Research Council.

### REFERENCES

Berman, C.—(1951) 'Primary Carcinoma of the Liver.' London (H. K. Lewis).
Blacklock, J. W. S., Kennaway, E. L., Lewis, G. M. and Urquhart, M. E.—(1954) *Brit. J. Cancer*, **8**, 40.
Campbell, J. M. and Cooper, R. L.—(1955) *Chem. & Ind.* (*Rev.*), 64.
Clemmesen, J.—(1951) *Acta Un. int. Cancr.*, **7**, 24.
Cooper, R. L.—(1954) *Analyst*, **79**, 573.
*Idem* and Lindsey, A. J.—(1955) *Brit. J. Cancer*, **9**, 304.
Doll, R.—(1951) *Acta Un. int. Cancr.*, **7**, 39.
Eberhardt, J. L.—(1949) 'Survey of Housing and Family Conditions in Orlando Township.' Thesis, Univ. Witwatersrand.
Higginson, J.—(1951) *Cancer*, **4**, 1224.
Khanolkar, V. R.—(1951) *Acta Un. int. Cancr.*, **7**, 51.
Kouwenaar, W.—(1951) *Ibid.*, **7**, 61.
Öhngren, L. G.—(1933) *Acta Otolaryng.*, *Stockh.*, Supp. 19, 1.
Shapiro, M. P., Keen, P., Cohen, L. and de Moor, N. G.—(1955) *S. Afr. med. J.*, **29**, 95.
*Idem*, Keen, P., Cohen, L. and Murray, J. F.—(1953) *Brit. J. Cancer*, **7**, 45.
Watt, J. M. and Breyer-Brandwyk, M. G.—(1932) 'The Medicinal and Poisonous Plants of Southern Africa.' Edinburgh (E. & S. Livingstone).
Wedgwood, P. and Cooper, R. L.—(1953) *Analyst*, **78**, 170.
Wynder, E. L., Graham, E. A. and Croniger, A. B.—(1953) *Cancer Res.*, **13**, 855.